# Efficacy and safety of baricitinib in patients with refractory alopecia areata

**DOI:** 10.1111/dth.15845

**Published:** 2022-10-18

**Authors:** Yuqian Wang, Taoming Liu, Sheng Li, Shunli Tang, Peiyi Lin, Yuwei Ding, Qingmiao Sun, Dingxian Zhu, Jianjun Qiao, Hong Fang

**Affiliations:** ^1^ Department of Dermatology, The First Affiliated Hospital Zhejiang University School of Medicine Hangzhou People's Republic of China

Dear Editor,

Alopecia areata (AA) is an autoimmune disorder characterized by nonscarring hair loss.^1^ Numerous treatment options are available for AA.^2^, ^3^ However, it is often challenging and unsatisfactory for refractory AA treatment. Recently, Janus kinase inhibitors (JAKis) have shown promising results in treating AA.^4^ For example, baricitinib has shown good clinical efficacy in two phase 3 trials involving patients with severe AA.^5^ Here we present a case series of 11 patients with refractory AA treated with baricitinib.

The Ethics Committee of The First Affiliated Hospital, Zhejiang University School of Medicine (Approved number: IIT2022089) approved this study. The following clinical characteristics were collected: age, sex, age at AA onset, disease duration, clinical type, previous treatment, and comorbidities. At each visit, symptom‐directed physical examination and clinical laboratory tests were conducted. All patients received at least one therapy for more than 1 year, including minoxidil, oral/topical/intralesional steroid, phototherapy, acupuncture, or herbal medicine prior to the initiation of baricitinib; however, the response was poor. All patients received baricitinib 2 mg twice daily for 20 weeks. Meanwhile, patients also used topical minoxidil as adjuvant therapy. The Severity of Alopecia Tool (SALT) was used to assess alopecia severity. SALT25, SALT50, and SALT75 were defined as 25%, 50%, and 75% regrowth, respectively. The results were divided into the following three categories: (I) “complete response” (CR), hair growth coexisted with no obvious plaques of hair loss; (II) “partial response” (PR), hair growth coexisted with obvious plaques; and (III) “no response” (NR), no hair growth. Adverse events were also recorded.

The results are summarized in Table 1. The subtypes of AA included alopecia universalis in two patients, alopecia totalis in two patients, multiple AA in six patients, and alopecia ophiasis in one patient. The median age at the beginning of AA was 17 (range, 3–14) and the median baseline SALT score was 54% (range, 32%–100%). Seven patients (64%) had nail involvement and five (45%) patients had total or partial body hair loss.

**TABLE 1:** Summary of patient characteristics

Patient	Age/sex	Age at AA onset (years)	Disease duration (months)	Clinical type	Previous treatment	SALT score before treatment	SALT score after treatment	Comorbidity	IL‐6	IL‐17A
1	24/F	17	85	MAA	DPCP, TS, SS	45	10	Thyroid disease	0.3	0.1
2	27/F	22	60	AT	TS, UV	87	4	Thyroid disease	0.1	0.1
3	24/F	22	27	AU	TS, ILC	100	26	NO	0.1	0.1
4	17/M	16	13	MAA	TS, SS, DPCP	42	14	NO	4.3	92.7
5	28/F	3	300	AU	TS, UV	100	100	Thyroid disease	3.2	28.3
6	29/F	26	37	AT	TS, UV	78	3	Thyroid disease	4.3	38.7
7	43/F	41	15	MAA	TS, SS	58	15	Psoriasis	5.7	87.2
8	22/M	18	52	MAA	TS, UV	54	50	Thyroid disease	0.1	0.1
9	18/M	7	133	Ophiasis	TS, UV, SS	32	15	NO	21.2	39.5
10	31/M	30	12	MAA	TS, UV	46	12	NO	2.2	63
11	15/M	14	15	MAA	TS, UV	41	41	NO	0.1	0.1

Abbreviations: AA, alopecia areata; AT, alopecia totalis; AU, alopecia universalis; DPCP, diphencyprone; F, female; ILC, intralesional corticosteroid; M, male; MAA, multifocal alopecia areata; SALT, Severity of Alopecia Tool; SS, systemic corticosteroids; TS, topical corticosteroids; UV, ultraviolet irradiation.

At week 20, two patients (18%) achieved CR, six patients (55%) achieved PR, and three patients (27%) were evaluated as NR. Nine patients responded positively to baricitinib treatment, one of whom subsequently lost regenerated hair, while the other two patients experienced no regrowth. The median SALT score was 15% (range, 3%–100%) and the median percent change in SALT score was 34% (range, 0%–83%) at week 20. Our results showed that seven patients (64%), three patients (27%), and two patients (18%) achieved SALT25, SALT50, and SALT75, respectively, while none of them achieved complete hair regrowth. Representative photos of two patients who had a typical response to baricitinib therapy are presented in Figure 1.

**FIGURE 1:**
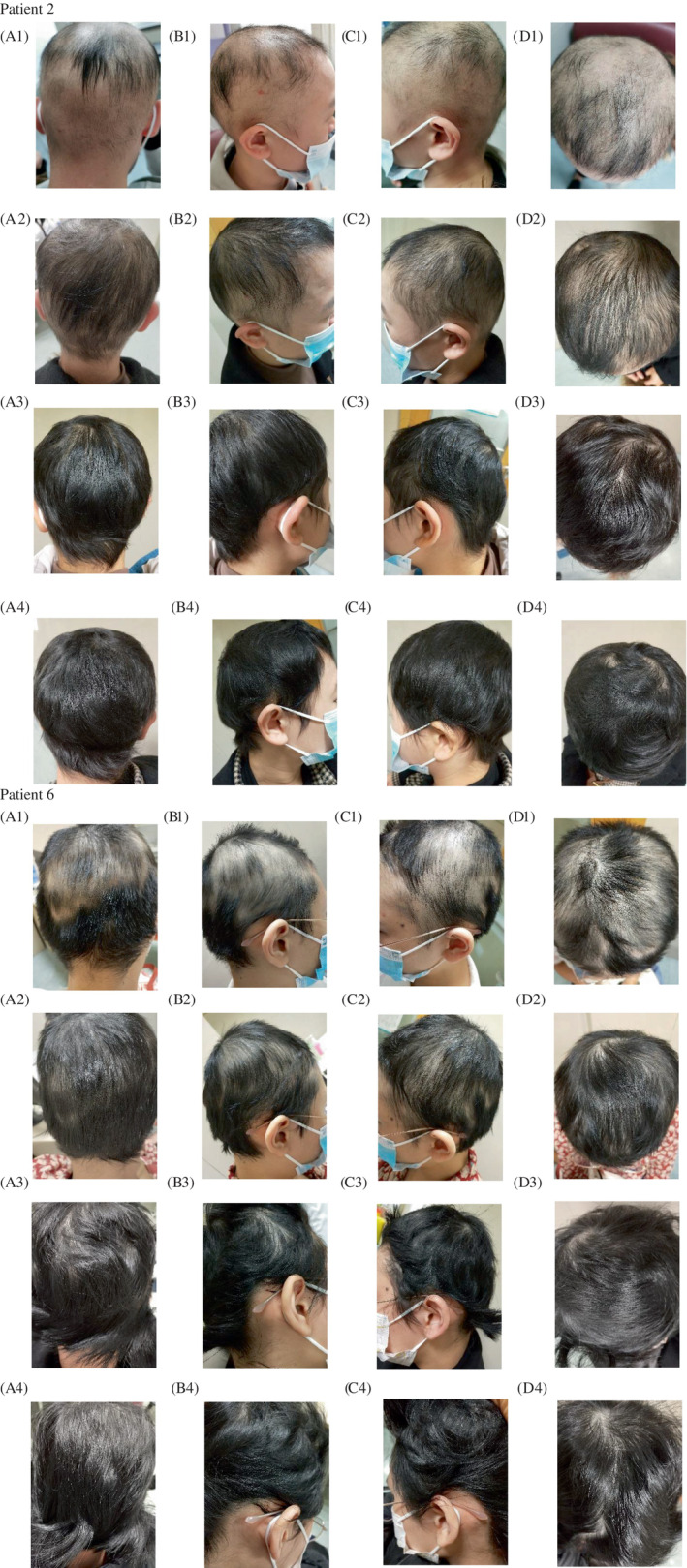
Clinical images of the “patient 2” and “patient 6” at 0–8‐12 and 20th weeks of follow‐up in the pictures 1–2–3‐4, respectively (a. Occipital view, b. Right parietal view, c. Left parietal view, and d. Posterior view)

Adverse events were mild and limited to nausea (*n* = 1), folliculitis (*n* = 2), and mild increases in liver transaminase levels (*n* = 1).

A meta‐analysis estimated that 72.4% of patients with AA responded to JAKis.^6^ Furthermore, one phase 2 trial^7^ and two phase 3 trials^5^ demonstrated that baricitinib 4 mg once daily proved beneficial over the 2‐mg dose. In our study, 27% achieved SALT50 with twice‐daily oral baricitinib at a 2‐mg dose, which is a remarkable finding because most patients had refractory AA. This series included only Chinese patients and they received a different dose of baricitinib because some patients were unable to tolerate the gastrointestinal reactions at a 4‐mg dose once daily. Further investigations are needed in the future to explore whether this difference in baricitinib response is due to ethnic or environmental differences. Moreover, numerous studies described an increased serum level of IL‐6 and IL‐17A in patients with AA.^8^, ^9^ Indeed, serum level of IL‐6 and IL‐17A was significantly elevated in 54.5% of all patients at the baseline in our study. After treatment with baricitinib, both levels returned to normal.

In summary, in our study, baricitinib is efficacious in refractory AA treatment. To determine the safety and efficacy of baricitinib for AA, further trials are warranted.

## AUTHOR CONTRIBUTIONS

*Study design*: Yuqian Wang, Jianjun Qiao, Hong Fang. *Data collection*: Yuqian Wang, Taoming Liu, Sheng Li, Shunli Tang, Peiyi Lin, Yuwei Ding. *Statistical analysis*: Yuqian Wang. *Data interpretation*: Qingmiao Sun, Dingxian Zhu. *Manuscript preparation*: Yuqian Wang, Jianjun Qiao, Hong Fang.

## FUNDING INFORMATION

This study was supported by the National Natural Science Foundation of China (81972931 and 81673045 to HF).

## CONFLICT OF INTEREST

The authors have no conflicts to disclose in relation to the present study.

## Data Availability

The data that support the findings of this study are available on request from the corresponding author. The data are not publicly available due to privacy or ethical restrictions.
